# Dynamics and Rheological Behavior of Chitosan-Grafted-Polyacrylamide in Aqueous Solution upon Heating

**DOI:** 10.3390/polym12040916

**Published:** 2020-04-15

**Authors:** Mengjie Wang, Yonggang Shangguan, Qiang Zheng

**Affiliations:** MOE Key Laboratory of Macromolecular Synthesis and Functionalization, Department of Polymer Science and Engineering, Zhejiang University, Hangzhou 310027, China; mengjiewang@zju.edu.cn (M.W.); zhengqiang@zju.edu.cn (Q.Z.)

**Keywords:** chitosan-grafted-polyacrylamide, thermo-thickening, rheological, dynamic light scattering, cryo-electron microscope

## Abstract

In this work, the transformation of chitosan-grafted-polyacrylamide (GPAM) aggregates in aqueous solution upon heating was explored by cryo-electron microscope (cryo-TEM) and dynamic light scattering (DLS), and larger aggregates were formed in GPAM aqueous solution upon heating, which were responsible for the thermo-thickening behavior of GPAM aqueous solution during the heating process. The heating initiates a transformation from H-bonding aggregates to a large-sized cluster formed by self-assembled hydrophobic chitosan backbones. The acetic acid (HAc) concentration has a significant effect on the thermo-thickening behavior of GPAM aqueous solution; there is a critical value of the concentration (>0.005 M) for the thermo-thickening of 10 mg/mL GPAM solution. The concentration of HAc will affect the protonation degree of GPAM, and affect the strength of the electrostatic repulsion between GPAM molecular segments, which will have a significant effect on the state of the aggregates in solution. Other factors that have an influence on the thermo-thickening behavior of GPAM aqueous solution upon heating were investigated and discussed in detail, including the heating rate and shear rate.

## 1. Introduction

Thermo-responsive polymers have been extensively studied over the past few decades owing to their industrial and biomedical applications [1,2,3,4,5,6,7]. Among them, thermo-thickening has received much attention from academic and industrial fields because of its huge application potential in many fields [3,7,8,9,10,11,12], especially in oil recovery [9,11]. It is always a great challenge in oil recovery to keep high viscosity of polymer displacement agent at a high temperature because the viscosity of most current oil displacement agents will decrease upon heating or in a high-temperature environment [9,11]. Differing from most of the commonly used polymers, which can hardly solve this problem [7,13], the thermo-thickening polymers whose viscosity can be enhanced during heating process have great potential to be used in enhanced oil recovery as well as water treatment and paper manufacturing [14]. In addition, because the thermo-thickening polymers with high concentration usually present a sol–gel transition temperature, they also have great potential in controlled permeation [7], tissue engineering [13], drug delivery [2,6], and so on.

Chitosan (CS) has received tremendous interest owing to its intrinsic properties, biocompatibility, nontoxicity, amphipathic, accessibility, and abundance in nature [15,16]. It is the precursor for other applications such as drug delivery [17,18,19], emulsifier [20], water treatment [21,22,23,24], and so on. Furthermore, it was found that CS modified by chemical or physical methods [25,26] could present thermo-thickening behavior and can be roughly divided into two kinds: CS complexes [27,28,29] and the derivatives of CS [30,31,32]. For CS complexes, thermo-thickening behaviors were less reported thus far except chitosan/poly(vinyl alcohol) (CS/PVA) [27] and chitosan/glycerophosphate (CS/β-GP) [29]. For CS derivatives, modification by poly-*N*-isopropylacrylamide (PNIPAM) is a common way to realize thermo-thickening [13,31], and recently, the carboxymethyl chitin also shows a novel thermo-thickening, which is pH- and temperature-dependent [33]. As there are many kinds of molecular interactions involved in these aqueous solutions mentioned above, it is difficult to elucidate the molecular mechanism of the thermo-thickening behavior of these complex systems. For CS/PVA, Schuetz et al. [27] thought that CS linked with PVA through hydrogen bonds at a low temperature. With temperature increasing, hydrogen bonds were broken and hydrophobic association among hydrophobic segments of CS chains gradually increased, as a result the gel-like structure formed. In CS/β-GP, there is no clear graph presented just excluding the influence of the hydrogen bond [29]. Among CS derivatives, the mechanism of thermo-thickening for modification by PNIPAM has been acknowledged [13,31,34] universally. Owing to the lower critical solution temperature (LCST) of PNIAPM, a phase separation will happen once the temperature rises to ~33 °C. Recently, the carboxymethyl chitin also showed a novel thermo-thickening associated with pH-dependence [33]. However, the mechanism is also not clear. By far, except CS-*g*-PNIPAM, there is no straight evidence to illustrate their assumption or figure out the mechanism.

Chitosan-grafted-polyacrylamide (GPAM) is one of the CS derivatives that has been reported to be a high-efficiency flocculating agent [35,36,37,38] and a potential oil-displacing agent [39]. Recently, we observed the thermo-thickening behavior of GPAM aqueous solution and proposed a preliminary outline of the molecular mechanism based on nuclear magnetic resonance (NMR) and transmission electron microscope (TEM) results. It was found the transformation from a hydrogen bonding (H-bonding) aggregate to a hydrophobic aggregate upon heating was responsible for the thermo-thickening [12]. However, some details during the thermo-thickening of GPAM solution are still unclear, such as the effect of acids on thermo-thickening, among others. In this work, we focus on the dynamics and rheological behavior of GPAM aqueous solution upon heating. GPAM samples with various grafting ratios, as shown in Table S1, are used to explore the thermo-induced structure in aqueous solutions using dynamic light scattering (DLS) and cryo-electron microscope (cryo-TEM). The influences of acid concentration, ramp rate, and shear rate on thermo-thickening of GPAM aqueous solutions are addressed.

## 2. Experimental Section

### 2.1. Materials

Chitosan (CS) powder and acetic acid-d_4_ (99.9%) were purchased from Sigma and Aldrich, Shanghai, China. Acetone (99.5%) and sodium hydroxide (96.0%) were purchased from Sinopharm Chemical Reagent Co., Ltd., Shanghai, China. Ammonium ceric nitrate (CAN, 99.0%), acetic acid (98.0%), acrylamide (99.0%), and deuterium oxide (D_2_O, 99.0%) were purchased from Aladdin, Shanghai, China. All chemicals and reagents were used without further purification.

### 2.2. Sample Preparation

Highly deacetylated chitosan was obtained by intermittent alkali treatment. Twenty (20) grams (g) of CS with a deacetylation of 78.9% was added into a mixture of distilled water (600 mL) and sodium hydroxide (300 g), which was stirred for 20 min at 110 °C. Then, the mixture was heated to 110 °C with mechanical agitation. After 1 h of mechanical agitation, the mixture was cooled down to room temperature and the precipitates were filtrated and washed by ultrapure water to neutrality. The chitosan, after being washed in water, was treated again in the alkaline solution for further deacetylation. The collected precipitate was dried at 50 °C until reaching a constant weight. The deacetylation degree of CS was increased by the intermittent alkali treatment to 98.0%, as determined by proton nuclear magnetic resonance (^1^H-NMR) using a Bruker 500 spectrometer (500 MHz) (Bruker, Karlsruhe, Germany) at room temperature (see Figure S1 in Supporting Information), and the calculation method of the degree of deacetylation is shown in Equation S1 in Supporting Information. The synthesis of chitosan-*g*-polyacrylamide (GPAM) has been presented in the previous work [12]. The graft ratio of GPAM was characterized by ^1^H-NMR (see Figure S2 in Supporting Information), and the calculation method of the graft ratio is shown in Equation S2. In this work, we synthesized GPAM samples with three kinds of grafting rate, as shown in Table S1.

### 2.3. Rheological Measurements.

Rheological experiments were performed on a stress-controlled rotational rheometer, Discovery Hybrid Rheometer-2 (DHR, TA Instruments, Newcastle, DE, USA). A 40 mm cone-plate geometry with a 2° cone angle and a 50 mm gap size was chosen for the steady shear tests. A 40 mm parallel plate geometry with a 500 mm gap size was chosen for all of the dynamic rheological tests. A defined amount of sample solution was directly poured very slowly onto the Peltier region in order to avoid the shear thinning effect and small air bubbles caused by using pipettes. Liquid paraffin was coasted around the margin of the solution sample to prevent the evaporation of solvent. Oscillatory temperature sweep tests were carried out using a strain amplitude of 0.2% (within the linear viscoelastic region, LVR), an oscillatory frequency of 6.283 rad·s^−1^, and a heating rate of 5 °C/min, unless otherwise stated.

### 2.4. Dynamic Light Scattering (DLS) and Ultraviolet and Visible Spectrum (UV)

The hydrodynamic radius *R*_h_ and size distribution were measured by dynamic light scattering (DLS) at the scattering angle of 90° using a 90 plus particle size analyzer (Brookhaven Instruments Corp., Holtsville, NY, USA) The wavelength of laser light was 635 nm. The CONTIN program was used for the analysis of dynamic light scattering data. The sample was filtered through the Millipore filters of 1 μm and 0.45 μm, successively, which was repeated at least three times.

The transmittance of GPAM solution was analyzed by a Lambda 35 UV/vis absorption spectrometer (PerkinElmer, Waltham, MA, USA) at 20 °C.

## 3. Results and Discussion

### 3.1. Transformation of GPAM Aggregates upon Heating

The structural transformation of GPAM in the solution sample upon heating was investigated by TEM and DLS in our previous work [12]. However, the microstructures of GPAM aqueous solution observed by TEM, which were obtained from dried solution samples, could not be the same as those in water. In addition, the concentration of GPAM used for DLS in the previous work is too low to observe the thermo-thickening process, because DLS is not appropriate to investigate high concentration polymer solution samples. Considering the possible impact of the above facts, in this work, we made appropriate improvements to investigate the macromolecular mechanism of thermo-thickening. Compared with ordinary TEM, the sample preparation method for cryo-TEM is to rapidly freeze the solution sample with liquid ethane, and then observe the frozen sample under low temperature and vacuum conditions [40,41]; as a result, the true structural form of GPAM in solution is preserved to the greatest extent. So, we used cryo-TEM instead of TEM to examine the structure of GPAM in aqueous solution. In addition, the transformation of GPAM aggregates in solution upon the heating process was investigated by increasing the concentration of GPAM solution used for DLS measurement while guaranteeing the existence of the thermo-thickening phenomenon.

**Figure 1:**
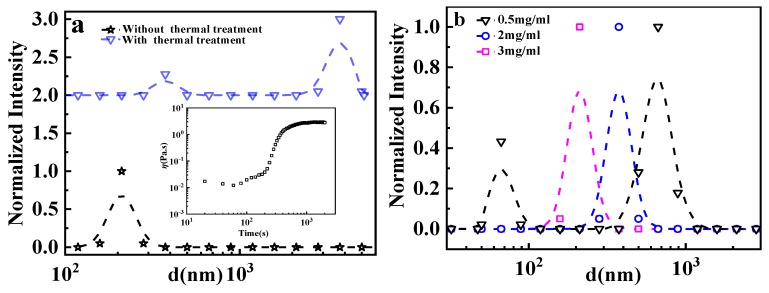
(**a**) Size distribution at 10 °C of 3 mg/mL chitosan−grafted−polyacrylamide 1 (GPAM1) solutions (0.01 M HAc) without and with a thermal treatment of holding at 40 °C for 3 min; inset displays the viscosity evolution of 3 mg/mL GPAM1 solutions with 0.01 M HAc at 40 °C. (**b**) Dependence of size distribution for GPAM1 solutions with 0.01 M HAc on concentration at 10 °C.

As the viscosity of the GPAM solution may start to increase when the temperature is higher than 20 °C, as reported previously [12], here, we investigate the macromolecular architecture in solution at 10 °C. To understand the evolution of the macromolecular architecture of GPAM upon heating, a thermal treatment at 40 °C for 10 min was applied to the solution sample. In addition, the thermo-thickening curve of the GPAM solution sample held at 40 °C was also investigated for comparison, as shown in the inset of Figure 1a. It can be observed that the viscosity of the solution sample basically tends to constant value after 10 min at 40 °C. As no thermo-thickening appears at 10 °C, the size distribution of GPAM is shown in Figure 1a. Only a single peak at about 200 nm appears for the original GPAM solution at 10 °C, which should be attributed to the aggregations of serval macromolecules rather than a single chain. After the sample was heated and held at 40 °C for 10 min, a bimodal distribution arises at 10 °C; a peak of about 400 nm and a peak of about several thousand nm. These results suggest that the aggregates with larger size form upon heating compared with the original sample.

It should be pointed out that the size distribution of GPAM aggregates in the initial sample measured here is about several hundred nanometers, and more aggregations with larger size appear during the thermo-thickening process. These results are because of the fact that we use a higher solution concentration of 3 mg/mL, which is closer to the truth of the GPAM molecular mechanism of the thermo-thickening process, rather than using a lower concentration solution to demonstrate the conformation and aggregation changes of GPAM molecules in the previous report [12]. Figure 1b gives the aggregation information of GPAM at different concentrations. When the concentration is low enough, about 0.5 mg/mL, it shows a bimodal distribution. The peak with a smaller size distribution, about ~80 nm, corresponds to single chain conformation, and the peak with a larger size, about ~700 nm, corresponds to macromolecular aggregates. In the cellulose solution and other solutions, the double peaks distribution was also reported [42]. With the increase of concentration, the single chain conformation disappears and the size of aggregations becomes smaller.

**Figure 2:**
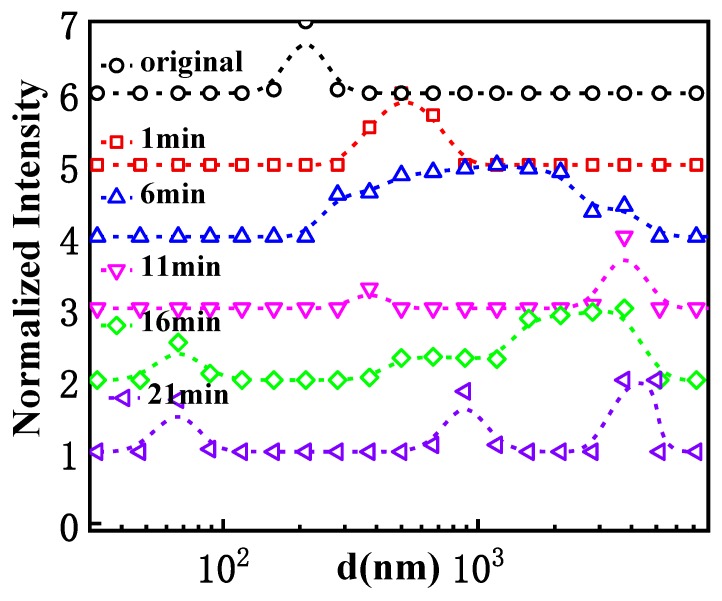
Size distribution of 3 mg/mL GPAM1 solutions with 0.01 M HAc at different times at 40 °C.

The size evolution of GPAM in aqueous solution at 40 °C is given in detail in Figure 2. The size distribution of GPAM always presents a single peak, while the size and peak width increase gradually until 6 min, indicating the formation of larger aggregations. After 11 min, the peak gradually evolves into a bimodal distribution; a larger single peak at about 3000 nm and a small peak at about 350 nm. The result is in accordance with Figure 1a. As time goes by, another smaller single peak at about 70 nm appears at 16 min and 21min, which corresponds to the single chain size. This is a very interesting result, because it means that some molecules not only do not contribute to thickening, but exist in the solution as single molecules. This indicated that the newborn lager aggregations upon heating were unstable with the increasing thermal treatment time; when the size of the association continues to increase, a small number of GPAM molecules are separated from the aggregates owing to the damage of hydrogen bonding, and exist as single molecules When the time reaches 21 min, there are even three peaks in the GPAM solution, indicating the macromolecular do exist in complex and heterogeneous forms during thermo-thickening.

During ordinary TEM sample preparation, there is a process of volatilization of the solvent and the solute in the solution will accumulate, so it is necessary to use a lower concentration sample for observation. The GPAM samples for cryo-TEM observation are obtained by rapidly freezing with liquid ethane, so it could realistically show the morphological feature of GPAM aggregates in solution. When the sample concentration is very low, the target content in the observation field is so low that it is difficult to observe. When the GPAM concentration reaches 10 mg/mL, the viscosity is relatively large, which is not conducive for sample preparation; therefore, we selected 6 mg/mL GPAM aqueous solution with 0.02 M HAc for cryo-TEM observation.

**Figure 3:**
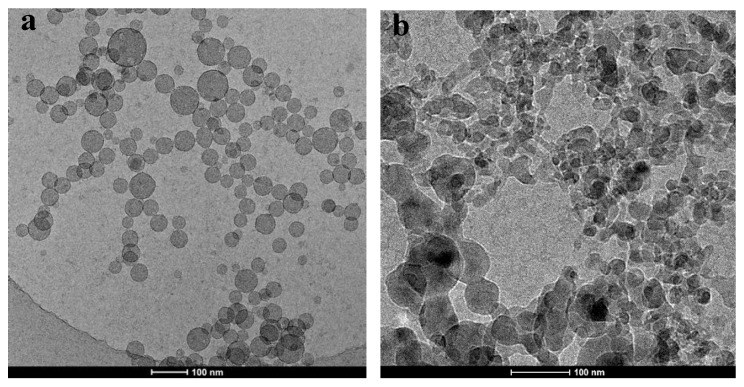
Cryo−electron microscope (cryo−TEM) observations of 6 mg/mL GPAM2 in 0.02 M HAc solution at (**a**) 10 °C and (**b**) 40 °C.

Figure 3a,b are the cryo-TEM images of the samples of GPAM solutions aged for 30 min at 10 °C and 40 °C respectively. The cryo-TEM images exhibit a dark domain and a sparse dark region. We simply consider the dark domain as aggregates and the sparse dark region as loose structures. Compared with Figure 3a, these aggregates gathered together and formed a much larger cluster structure. This result is consistent with the results measured by DLS above and confirms the formation of larger-size aggregates in GPAM solution during the heating process.

### 3.2. Effects of HAc on GPAM Solution

As GPAM aqueous solution in the presence of HAc, the effect of HAc on the existence of GPAM must be considered. When GPAM is dispersed in acetic acid solution at different concentrations, its ionization in water will obey the following law:

(1) $$GPAM-NH_{2}+HAc\overset{K}{\to }GPAM-NH_{3}^{+}+Ac^{-}$$

Usually, the pKa value of CS is about 6.5 [43], and the pka value of HAc is about 4.76 [44]:

(2) $$K=\frac{\left[-NH_{3}^{+}\right]\left[Ac^{-}\right]}{\left[-NH_{2}\right]\left[HAc\right]}=\frac{\left[Ac^{-}\right]\left[H^{+}\right]}{\left[HAc\right]}\times \frac{\left[-NH_{3}^{+}\right]}{\left[-NH_{2}\right]\left[H^{+}\right]}=\frac{Ka_{HAc}}{Ka_{CS}}$$

According to Equation (1) and (2), the relationship between the degree of protonation of the amino group and the concentration of HAc can be obtained, as shown in Figure S3 in Supporting Information. As the concentration of HAc increases, the degree of protonation of the amino group increases.

**Figure 4:**
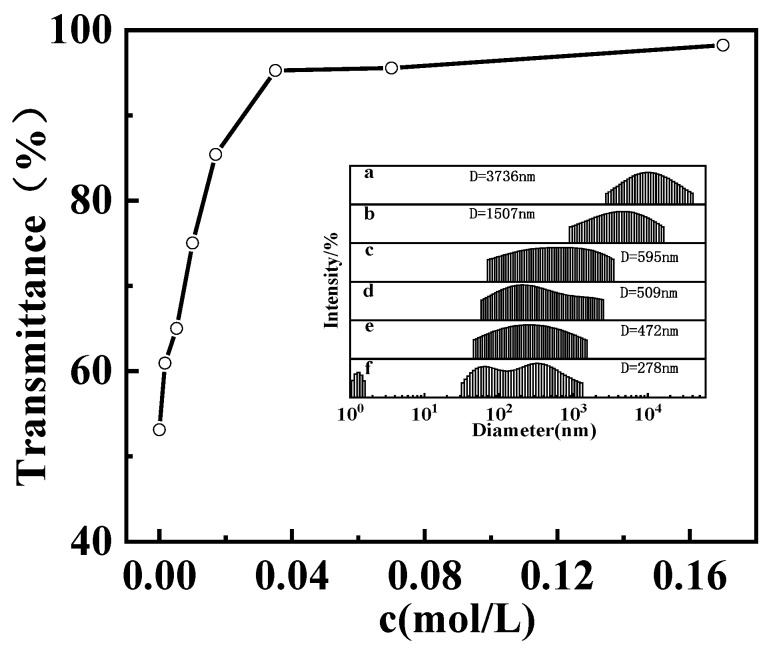
Optical transmittance at 600 nm for 10 mg/mL GPAM2 aqueous solutions as a function of HAc concentration at 20 °C. Inset displays the particle size of 10 mg/mL GPAM2 aqueous solutions with the HAc concentration of (a) 0.00175 M, (b) 0.0105 M, (c) 0.0175 M, (d) 0.0351 M, (e) 0.0702 M, and (f) 0.175 M.

Figure 4 gives the transparency for the GPAM solution with the increasing HAc concentration. It is stable when the concentration of HAc is higher than 0.035 M. The macromolecules and aggregates in GPAM solution decrease gradually with the increase of HAc concentration, as shown in the inset of Figure 4. This is because the protonation of –NH_2_ is enhanced with the increasing HAc; the electrostatic repulsion between GPAM molecular segments increases accordingly; and, consequently, the hydrogen bond interaction among aggregations is gradually weakened, resulting in a decrease of the aggregation’s size.

**Figure 5:**
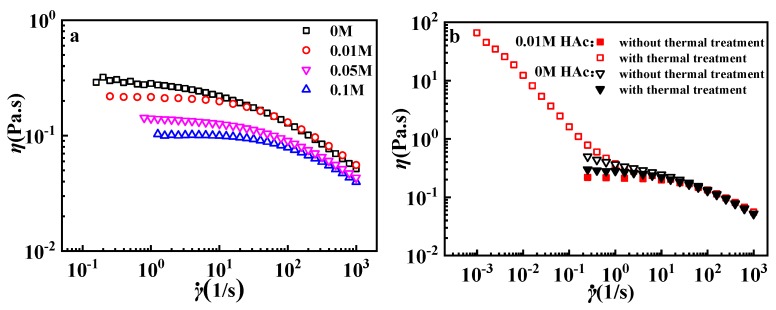
Steady flow curves of 10 mg/mL GPAM2 solutions (**a**) with different HAc concentration and (**b**) with and without a thermal treatment (holding at 60 °C for 15 min). All tests were conducted at 10 °C.

Figure 5a gives the steady flow results of GPAM at different HAc concentrations. All samples show obvious shear thinning. With the increase of HAc, the viscosity of GPAM solutions decreases, especially at a low shear rate. All GPAM solution samples present a constant viscosity in the low shear rate region, so zero-shear-rate viscosity (*η*_0_) can be obtained from steady flow curves, as listed in Table 1. *η*_0_ decrease with the increasing concentration of HAc indicates the decrease of the aggregation’s size, which is in accordance with the above results. However, irreversible thermo-thickening can be found in the GPAM solution with 0.01 M HAc after a thermal treatment (holding at 60 °C for 15 min) rather than the GPAM solutions without HAc. As shown in Figure 5b, the GPAM solution presents a slight increase of viscosity at a low shear rate after thermal treatment, while viscosity for the GPAM solution with 0.01 M HAc increases sharply at a low shear rate. Those results indicate that HAc plays an important role in the thermo-thickening behavior of the GPAM solution.

**Figure 6:**
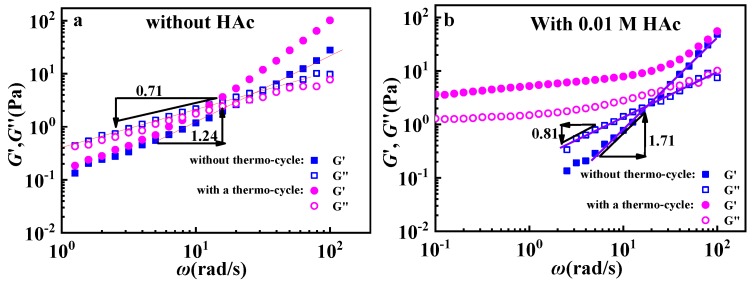
Frequency sweeps of 10 mg/mL GPAM2 solutions (**a**) without HAc and (**b**) with 0.01 M HAc subjected to thermo−cycle (temperature increases from 10 °C to 60 °C, then decreases to 10 °C, ramp rate: 5 °C /min) at 10 °C.

To further investigated the effect of acid on the structure evolution of the GPAM solution during the above thermo-cycle, frequency sweeps for GPAM solutions subjected to thermal cycle were conducted at 10 °C. In Figure 6a, moduli for the two GPAM solutions without HAc have the similar tendency: *G*′ lower than *G*″ in the low frequency regime and *G*′ larger than *G*’’ in the high frequency regime, while their G″ are close in the whole investigation range. In general, *G*′ ~ *ω*^2^ and *G*’’ ~ *ω* mean the existence of a homogenous structure; the decreased exponent indicates the existence of a physical network or aggregations [27]. In Figure 6a, the exponent is smaller than the theoretical value, indicating the existence of aggregations in the GPAM solution without HAc in a sense. Figure 6b gives the frequency sweep results for solution samples containing HAc. When the sample is subjected to thermo-cycle, *G*′ is larger than *G″* in the low frequency regime and the modules present a weak frequency dependency. This suggested a sol-to-gel transformation had taken place. On the contrary, the GPAM solutions without thermo-cycle also seem sol-like. Therefore, GPAM solution containing HAc presents an irreversible structure evolution from aggregations to a more structured fluid in the thermo-cycle for GPAM solutions.

**Figure 7:**
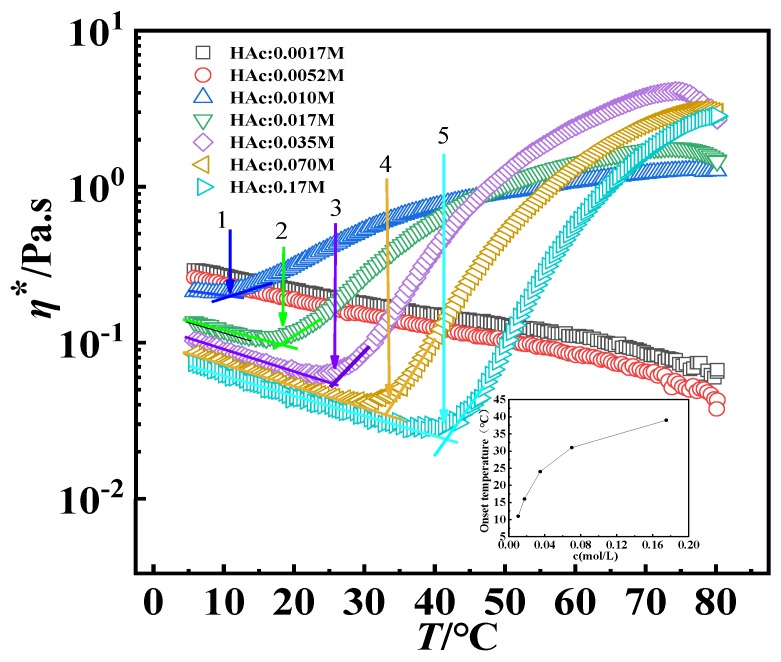
Influence of HAc concentration on thermo−thickening for the GPAM aqueous solution with 10 mg/mL concentration. Inset displays the relationship between onset temperature and concentration of HAc in the 10 mg/mL GPAM aqueous solution. The strain is 0.2% and the oscillatory frequency is 6.283 rad·s^−1^. The heating rate of the samples is 5 °C/min.

The thermo-thickening process of GPAM solutions was investigated as a function of HAc concentration; Figure 7 gives the temperature sweep results of GPAM solution samples with different HAc concentrations and the mass concentration of the GPAM solution is fixed at 10 mg/mL. The different HAc concentrations determine the content of the protonation of –NH_2_, that is, in the range of the HAc concentration of 0.00175 M to 0.175 M, the larger the concentration of HAc, the larger amount of the protonation of –NH_2_ of the moiety. As shown in the inset of Figure 7, the onset temperature (the critical temperature at the onset of an increase in viscosity) increases with the concentration of HAc. This may be attributed to stronger hydrophilicity and electrostatic repulsion induced by more protonation of –NH_2_, whichs inhibit the hydrophobic aggregation. The onset temperature obviously decreases when the concentration of HAc decreases to 0.01 M. Moreover, both GPAM solutions containing 0.0017 M and 0.0052 M HAc present little thermo-thickening, indicating that there is a critical value of the concentration (>0.005 M) for the thermo-thickening of 10 mg/mL GPAM solution. This is concentration of HAc may be too low, leading to the protonation of GPAM being significantly less and a correspondingly small amount of positive charges, so the electrostatic repulsive force between GPAM molecules is very weak and the water solubility is poor, which leads to the shrinkage of molecular segments, and the molecules are tightly bonded through hydrogen bonding. These densely structured hydrogen-bonded associations cannot be destroyed easily during the heating process, so GPAM molecules cannot be reorganized in large-sized association structures through hydrophobic association, and the thermo-thickening behavior cannot be exhibited. When the HAc concentration reaches a certain value, the water solubility of GPAM increases and the electrostatic repulsion will destroy some of the intermolecular hydrogen bonds, so the structure of the GPAM association will be loose in the aqueous solution. The molecular chain can be extracted from the hydrogen-bonded association and form a large-sized association structure through hydrophobic association, thereby exhibiting thermo-thickening behavior. It is worth mentioning that when the HAc concentration is 0.01 M, the onset temperature of thermo-thickening is the lowest, which means the thermo-thickening structures can be formed easily; therefore, we chose the GPAM aqueous solution with 0.01 M HAc for the following rheological experiments.

### 3.3. Influence of Heating Rate and Shear Rate

**Figure 8:**
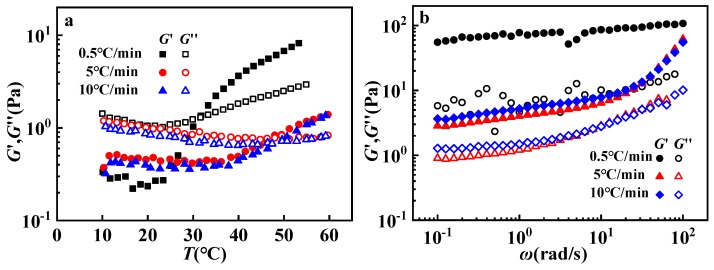
(**a**) Temperature sweeps of 10 mg/mL GPAM2 solutions with 0.01 M HAc during the heating process at different ramp rates; (**b**) frequency sweeps at 10 °C for 10 mg/mL GPAM2 solutions with 0.01 M HAc after a thermal cycle with different ramp rates (from 10 to 60 to 10 °C).

Figure 8a gives the influences of heating rate on the thermo-thickening behavior. The sample presents a lower *T*_trans_ (the temperature for *G*′–*G*″ crossover) at a slow heating rate, indicating *T*_trans_ has a dependence of heating rate. When the heating rate was 0.5 °C, *G′* and G″ of the GPAM aqueous solution after thermo-cycle treatment were significantly greater than those of 5 °C and 10 °C. These results indicate that a slower heating rate is conducive to the formation of thermo-thickening structures. The thermo-thickening behavior of the GPAM aqueous solution is dependent on the heating time and temperature; the thermo-thickening structure of the GPAM aqueous solution will be more complete at a longer heating time or a higher temperature [12]. When the heating rate is lower, it means that the heating time and the residence time in the high temperature region are longer, so the *T*_trans_ will be lower and G′, G″ will be greater. Furthermore, it can be found from Figure 8a,b that modulus variation with temperature and ω were consistent with each other for 5 °C/min and 10 °C/min. The possible reason may lie in that structure evolution cannot keep up with the ramp rate to cause no apparent discrepancy in a higher ramp rate.

**Figure 9:**
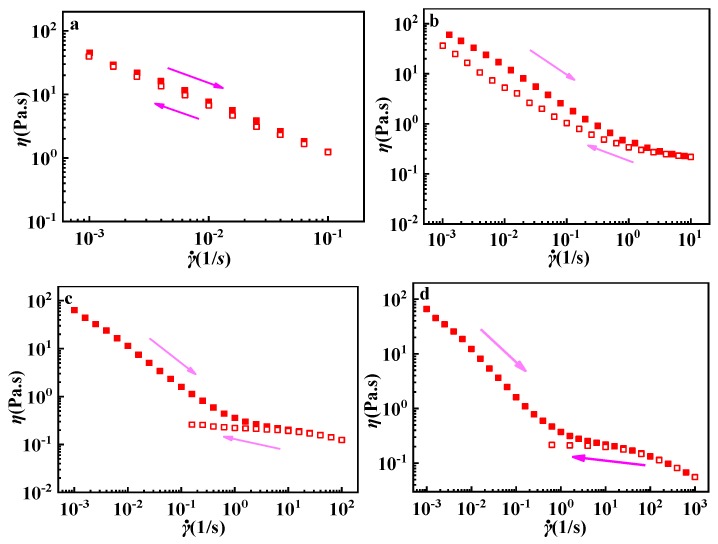
Shear and recovery rates of 10 mg/mL GPAM2 solutions with 0.01 M HAc in different limits of the upper shear rate of (**a**) 0.1 s^−1^, (**b**) 10 s^−1^, (**c**) 100 s^−1^, and (**d**) 1000 s^−1^, with a thermal treatment (holding at 60 °C for 15 min). All tests were conducted at 10 °C.

In order to study the shear-resistance of GPAM after thermo-thickening, a serious of shear recover experiments were carried out by fixing different maximal shear rates. As shown in Figure 9, the increased viscosity of the GPAM solution after thermo-treatment can remain. Furthermore, with the increase of shear rate, the viscosity shear thinning occurs, while its viscosity can recover well as the shear rate decreases in 0.1~0.001 s^−1^ (Figure 9a). With the shear rate range increasing, the viscosity cannot recover in time as the shear rate decreases in 10~0.001 s^−1^, meaning that the damaged associated structure of GPAM in solution needs more time to achieve complete recovery (Figure 9b). When the maximum shear rate reached 100 s^−1^ or 1000^−1^, the structure was broken thoroughly and came back to the original structure without thermal treatment (Figure 8c,d).

## 4. Conclusions

During the heating process, large-size aggregates were formed in the GPAM aqueous solution through hydrophobic association from the hydrophobic groups on GPAM, which were responsible for the thermo-thickening of the GPAM aqueous solution. As the concentration of HAc in the GPAM aqueous solution increased, the protonation degree of GPAM increased and the electrostatic repulsive force between GPAM molecules would gradually increase, so the size of the GPAM aggregates in the solution gradually decreased. When the concentration of HAc was less than 0.05 M, the protonation degree of GPAM was very low and the solubility was very poor, and then a dense hydrogen bonding association was formed in the solution, which cannot be destroyed during the heating process. As a result, the thermo-thickening behavior disappeared. Furthermore, a higher HAc concentration leads to stronger hydrophilicity and electrostatic repulsion induced by more protonation of –NH_2_, which inhibit the hydrophobic aggregation. In addition, a slower heating rate is conducive to the formation of thermo-thickening structures and a strong shear rate will destroy the thermo-thickening structure of GPAM.

## Figures and Tables

**Table 1:** *η*_0_ of 10 mg/mL chitosan-grafted-polyacrylamide 2 (GPAM2) solutions with different HAc concentration at 10 °C.

HAc (mol/L)	*η_0_* (Pa·s)
0	0.28
0.01	0.22
0.05	0.15
0.1	0.10

## Supplementary Materials

The following are available online at https://www.mdpi.com/2073-4360/12/4/916/s1, Figure S1: ^1^H-NMR spectrums of 0.5 wt.% CS solution in an acetic acid-d_4_/water-d_2_ mixture at room temperature (the DDA value of samples of (a) and (b) are 79% and 98% respectively). Figure S2: ^1^H-NMR spectrums of CS and GPAM in an acetic acid-d4/water-d2 mixture at room temperature. Figure S3: Relationship between the degree of protonation of the amino group and the concentration. Table S1: Molecular parameters of GPAM.
