# Supplementary material for: Dynamics and Rheological Behavior of Chitosan-Grafted-Polyacrylamide in Aqueous Solution upon Heating
Source: Polymers (Basel). 2020 Apr 15;12(4):916. doi: 10.3390/polym12040916 (PMC7240601; doi:10.3390/polym12040916)
Supplement: Supplementary file 1 [file polymers-12-00916-s001.pdf]

Supporting Information for

Dynamics and rheological behavior of Chitosan-grafted-polyacrylamide in aqueous solution upon heating

Mengjie Wang, Yonggang Shangguan, *, Qiang Zheng

MOE Key Laboratory of Macromolecular Synthesis and Functionalization, Department of Polymer Science and Engineering, Zhejiang University, Hangzhou 310027, PR China.

Proton nuclear magnetic resonance (1H-NMR)

Figure S1 gives the 1H-NMR spectrums of CS before and after deacetylation treatment. The peak area at 7 is recorded as A, and the peak area at 2-6 is recorded as B, then the calculation formula of DDA is as follows:

$$DDA(\%)=\left(1-\frac{\frac{1}{3}A}{\frac{1}{6}B}\right)\times 100\% = \left(1-\frac{2A}{B}\right)\times 100\% \qquad (1)$$

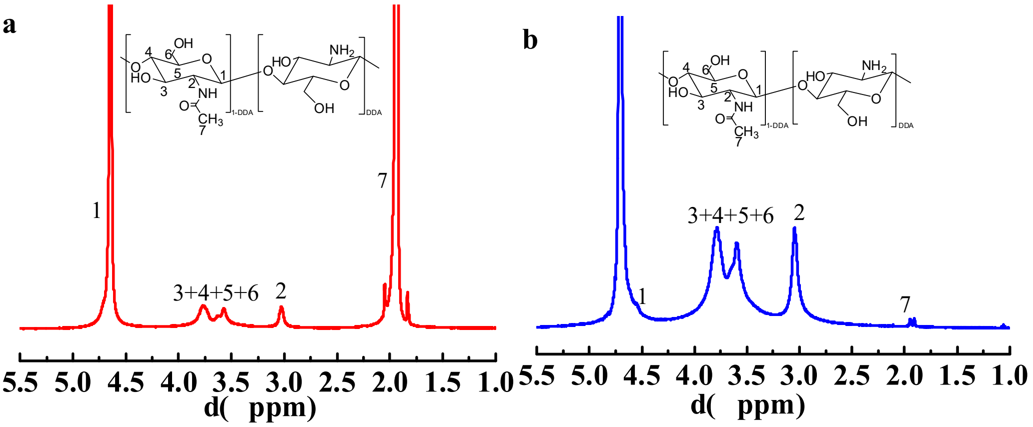

Figure S1 ^{1}H-NMR spectrums of 0.5 wt% CS solution in an acetic acid-d_4 /water-d_2 mixture at room temperature (the DDA value of samples of (a) and (b) are 79% and 98% respectively).

Figure S2 gives the 1H-NMR spectrums of CS and GPAM. The multiple peaks at 2.5 ppm and 1.5 ppm correspond to the chemical shifts of -CH- and -CH2-H of PAM, demonstrating the PAM chains were successfully grafted onto the CS backbone. Using the area ratio of the characteristic peaks of H, the grafting ratio of PAM can be calculated. The mass of the PAM chain is represented by W_{PAM}, the mass of the CS chain is represented by W_{cs}, the area of the multiple peak at 1.5 ppm is represented by C, the molecular amount of monomer AM is represented by M_{AM}, and the average molecular weight of one monomer unit in CS is represented by $M_{CS,monomer}$, then the formula is as follows:

$$G(\%)=\left(\frac{W_{PAM}}{W_{CS}}\right)\times 100\% = 3\times\frac{C}{B}\times\frac{M_{AM}}{M_{CS,monomer}}\times 100\% \qquad (2)$$

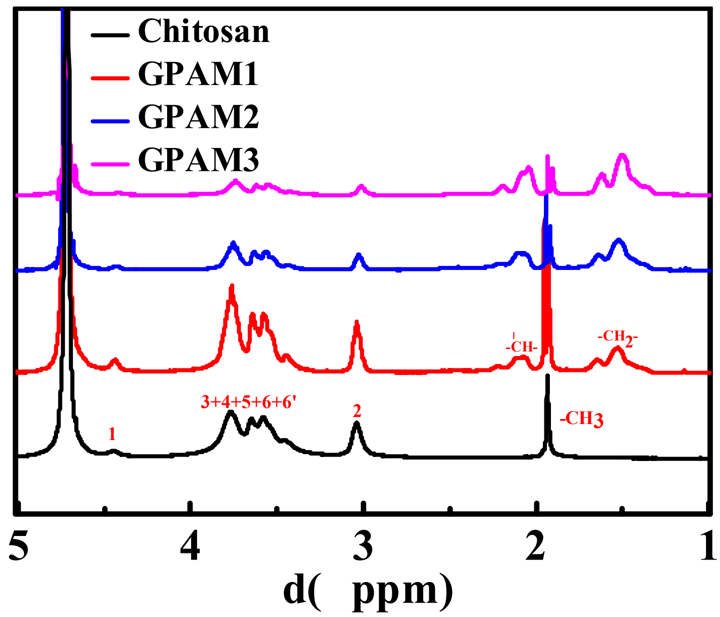

Figure S2 1H-NMR spectrums of CS and GPAM in an acetic acid-d4 /water-d2 mixture at room temperature.

With the help of 1H-NMR (Figure S2) the graft ratio could be calculated and were listed on Table S1.

Table S1 Molecular parameters of GPAM

Sample	Graft ratio (%)
GPAM1	24.6
GPAM2	101.5
GPAM3	201.0

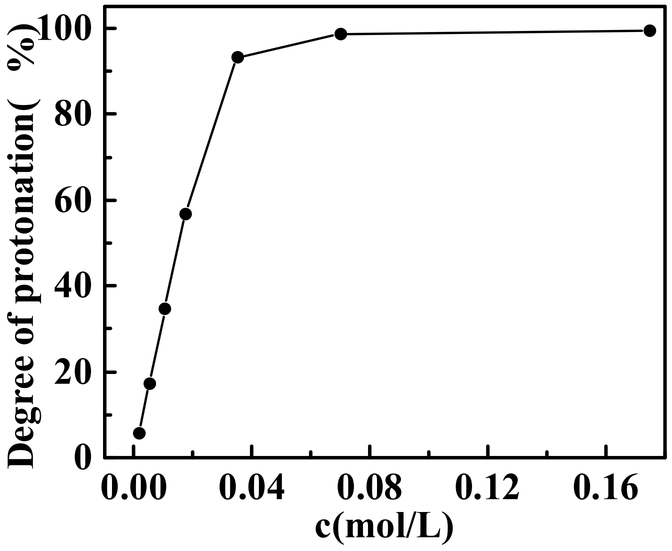

Figure S3 Relationship between the degree of protonation of the amino group and the concentration.
